# Plants as Factories for Human Pharmaceuticals: Applications and Challenges

**DOI:** 10.3390/ijms161226122

**Published:** 2015-12-02

**Authors:** Jian Yao, Yunqi Weng, Alexia Dickey, Kevin Yueju Wang

**Affiliations:** 1 Emergency Department, The Affiliated Hospital of Qingdao University, Qingdao 266003, China; yaojian2002@126.com (J.Y.); wengyunqi@126.com (Y.W.); 2 Department of Natural Sciences Northeastern State University at Broken Arrow, Broken Arrow, OK 74014, USA; dickey@nsuok.edu

**Keywords:** plant molecular farming, edible vaccine, humanized glycan, transient expression, seed platform

## Abstract

Plant molecular farming (PMF), defined as the practice of using plants to produce human therapeutic proteins, has received worldwide interest. PMF has grown and advanced considerably over the past two decades. A number of therapeutic proteins have been produced in plants, some of which have been through pre-clinical or clinical trials and are close to commercialization. Plants have the potential to mass-produce pharmaceutical products with less cost than traditional methods. Tobacco-derived antibodies have been tested and used to combat the Ebola outbreak in Africa. Genetically engineered immunoadhesin (DPP4-Fc) produced in green plants has been shown to be able to bind to MERS-CoV (Middle East Respiratory Syndrome), preventing the virus from infecting lung cells. Biosafety concerns (such as pollen contamination and immunogenicity of plant-specific glycans) and costly downstream extraction and purification requirements, however, have hampered PMF production from moving from the laboratory to industrial application. In this review, the challenges and opportunities of PMF are discussed. Topics addressed include; transformation and expression systems, plant bioreactors, safety concerns, and various opportunities to produce topical applications and health supplements.

## 1. Introduction

The production of plant-derived pharmaceuticals has attracted great interest. Mapp Biopharmaceutical Inc., a company located in San Diego, CA, USA has produced a drug in tobacco leaves called ZMapp, which has been used to combat the 2014 Ebola virus outbreak in Africa [1]. As of October 2014, seven infected patients received an early treatment with ZMapp and fully recovered. Another patient, receiving a late treatment with ZMapp in November 2014, however, succumbed to the disease and died. Additional Ebola patients were unable to receive the treatment due to an insufficient supply of ZMapp. This is unfortunate since it is the only drug to date that has been effectively used to treat patients infected with the Ebola virus, even though it has not been approved by the U.S. Food and Drug Administration (FDA). ZMapp has been subjected to clinical Phase I and 2 trials in 2015, sponsored by the National Institute of Allergy and Infectious Diseases (NIAID) (see Table 1). On 15 September 2015, ZMapp was granted a fast track status by the FDA [2]. Middle East respiratory syndrome coronavirus (MERS-CoV) is an emerging disease. Due to the high mortality rate of MERS (above 35%), it caused a public panic in South Korea during May 2015. As of 27 November 2015, MERS-CoV has infected 1618 patients and caused a total of 579 deaths worldwide. Over 26 countries have reported MERS-CoV cases [3]. Currently, no effective drug is available to treat the MERS-CoV virus. Plant Biotechnology Inc. (Hayward, CA, USA) produced an immunoadhesin (DPP4-Fc) in transgenic tobacco. Purified DPP4-Fc exhibits strong binding to MERS-CoV and prevents the virus from infecting lung cells. In June 2015, Plant Biotechnology Inc. received funding from NIAID to support further development and testing of this drug [4].

The concept of using plants to produce recombinant pharmaceutical proteins, referred to as plant molecular farming (PMF) or pharming (PMP), is not new. Human growth hormone, initially produced in tobacco and sunflower in 1986, was the first-plant-derived recombinant therapeutic protein [5]. Mason *et al.* [6] later expressed the hepatitis B surface antigen (HBsAg) in transgenic tobacco. This plant-derived antigen was physically and antigenically similar to the HBsAg obtained from human serum and recombinant yeast. The yeast-derived HBsAg is clinically used for HBV vaccination. Since 1994, more than 100 pharmaceutical proteins have been expressed and characterized in plants. By 2011, more than twenty PMF pharmaceuticals were placed in preclinical or clinical trials [7]. Several PMF products have completed Phase 2 trials and one product has been approved by the FDA (Table 1). Although several plant-derived drugs have been commercialized as research and diagnostic reagents (such as tobacco derived aprotinin and rice derived lysozyme from Sigma-Aldrich Company (St. Louis, MO, USA) or received USDA approval as a vaccine additive for use in poultry (Dow Agro Sciences, Indianapolis, IN, USA) [1], the current review mainly focuses on PMF in relation to human pharmaceutical applications. Plants represent a promising system for the production of human pharmaceutical proteins on a large scale, and at a low cost. Many production challenges, however, such as low yield [7,8,9,10], plant glycosylation [11,12,13], purification and downstream processing hurdles [14,15,16], have limited the development of PMF-based human pharmaceuticals on a clinical scale.

In May 2012, the first PMF-derived enzyme, ELELYSO™ (taliglucerase alfa) (Protalix BioTherapeutics, Karmiel, Israel), was approved for human use by the FDA [17]. ELELYSO™ is based on the use of carrot cells to produce recombinant taliglucerase alfa, which is used in an enzyme replacement therapy to treat adult patients with Gaucher disease. The production and application of ELELYSO™, however, is not representative of other PMF-derived pharmaceuticals for several reasons. Since Gaucher disease is a rare genetic disease, mostly found among Ashkenazi Jews, ELELYSO™ has limited production needs. The FDA also accelerated (fast tracked) the approval process as a treatment for a rare disease. Additionally, the drug is produced in carrot cells using a large bioreactor under very stringent conditions. This process is different from production of other PMF products, which generally use entire leaves, fruits, seeds, or whole plants to produce the recombinant pharmaceutical. The production and approval of ELELYSO™ still represents a major step forward for the whole field of PMF. Many companies have now explored and started product pipelines utilizing plant-expression systems (see Table 2).

**Figure 1:**
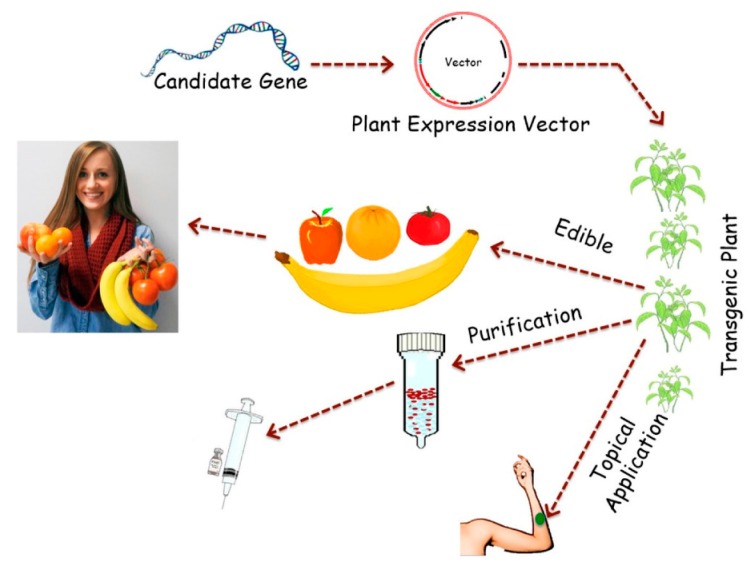
Diagrammatic illustration of the process of Plant Molecular Farming (PMF).

Identifying potential genes suitable for PMF and general approaches is becoming more simple and straight forward. Facilitated by the rapid progress in genomics, proteomics, and bioinformatics, a greater number of useful genes are being identified and characterized. Additionally, relatively routine molecular methods have become available for placing the genes of interest into plant expression vectors and transforming them into plants (see Figure 1).

**Table 1:** Examples of plant-derived pharmaceuticals in clinical trials (data from U.S. National Institutes of Health Clinical Trial [18].

Product	Host	Application	Clinical Trial	Status	Sponsor
Taliglucerase alfa; Recombinant glucocerebrosidase (prGCD)	Carrot cell culture	Gaucher disease	NCT00376168	Phase 3 completed (2012); FDA approved (2012)	Protalix, Karmiel, Israel
ZMApp	Tobacco	Ebola Virus	NCT02363322	Phase 1 and 2 (2015)	National Institute of Allergy and Infectious Diseases (NIAID), Bethesda, MD, USA
PRX-102	Tobacco cell culture	Fabry Disease	NCT01769001	Phase 1 and 2 (2014)	Protalix, Karmiel, Israel
VaccinePfs25 VLP	Tobacco	Malaria	NCT02013687	Phase 1 (2015)	Center for Molecular Biotechnology, Plymouth, MI, USA
Vaccine Recombinant protective antigen	Tobacco	Anthrax	NCT02239172	Phase 1 (2014)	Center for Molecular Biotechnology, Plymouth, MI, USA
HAI-05	Tobacco	H5N1 Vaccine	NCT01250795	Phase 1 (2011)	Center for Molecular Biotechnology, Plymouth, MI, USA
Recombinant human intrinsic factor	*Arabidopsis thaliana*	Vitamin B12 deficiency	NCT00279552	Phase 2 Completed (2006)	University in Aarhus, Aarhus, Denmark
H5-VLP + GLA-AF Vaccine	Tobacco	Influenza A Subtype H5N1 Infection	NCT01657929	Phase 1 Completed (2014)	Infectious Disease Research Institute, Seattle, WA, USA
P2G12 Antibody	Tobacco	HIV	NCT01403792	Phase 1 Completed (2011)	University of Surrey, Guildford, UK

**Table 2:** Examples of companies utilizing PMF to produce human pharmaceuticals (data from company websites).

Company	Host	Lead Product	Expression Technology	Advantage	Website References
Mapp Biopharmaceutical/LeafBiol, USA	Tobacco leaves	ZMapp™ for Ebola crisis	MagnICON Transient expression	Speed	[2]
Protalix, Carmiel, Israel	Carrot or tobacco cell culture	ELELYSO™ (taliglucerase alfa) Enzyme replacement	ProCellEx^®^ Stable Expression	Quality	[19]
Icon Genetics, München, Germany	*Nicotiana benthamiana* leaves	Vaccine for non-Hodgkin’s Lymphoma	MagnICON Transient expression	Speed and Personalization	[20]
Ventria Bioscience, Junction City, KS, USA	Rice seeds	VEN150 for HIV-associated chronic inflammation	Express Tec Stable Expression	Scale Cost	[21]
Greenovation Biotech GmbH, Heilbronn, Germany	Moss	Moss-GAA for Pompe Disease, Moss-GBA for Gaucher’s Disease, Moss-AGAL for Fabry Disease	Moss *Physcomitrella patens* based Broytechnolgy	Speed Scale and Customized	[22]
Kentucky BioProcessing, Owensboro, KY, USA	*Nicotiana benthamiana* leaves	Contract service	Geneware Transient expression	Speed	[23]
PhycoBiologics Inc. Bloomington, IN, USA	Algae	Vaccines Growth Factor and enzymes	Microalgae expression	Speed Scale	[24]
Medicago, Québec, QC, Canada	*Nicotiana benthamiana* Alfalfa	Vaccine for influenza, Pandemic market, Rabies and Rotavirus	Proficia™ Transient Expression; Stable Expression	Speed	[25]
Synthon, Nijmegen, The Netherlands	Duckweed LeafyBiomass	Antibody for non-Hodgkin’s Lymphoma	LEX system Stable expression	Speed Quality	[26]
Fraunhofer IME, Aachen, Germany	Tobacco leaves	HIV Antibody	Stable Nuclear Expression	Scale Cost	[27]
Fraunhofer CMB/iBio, Newark, DE, USA	*Nicotiana benthamiana* leaves	Influenza vaccine	Transient expression	Speed	[28]
Healthgen, Wuhan, Hubei, China	Rice seed	Serum albumin	Stable Expression	Quality Scale	[29]
PlanetBiotechnology, Hayward, CA, USA	Tobacco leaves	CaroRx for dental caries; PBI-220 antibody for anthrax; DPP4-Fc for MERS coronavirus infection	Stable Expression	Quality Scale	[4]

An example of an early proof of concept for PMF is the production of plant-derived edible human vaccines, using leafy plants or fruits [30,31,32,33,34,35]. Edible vaccines are an ideal product in concept since the vaccine could be administered to people orally, theoretically without the need of professional health care workers or sterile injections. The tedious and complicated process of purifying and storing the vaccine would also be eliminated since the food product itself would be the vaccine. The plants could also be grown locally, thus negating the cost of long distance transportation and storage. Edible vaccines also avoid the potential risk of infecting patients with a contaminated product since, in general, organisms causing plant diseases do not infect humans. The edible vaccine concept was first proposed by Charles Arntzen and coworkers, after HBsAg (Hepatitis B Virus antigen) was produced in tobacco plants. Mice fed HBsAg-transgenic potatoes exhibited a robust immune response [36]. Uncooked potatoes from transgenic potato plants producing HBsAg were later tested orally in humans. Greater than 60% of the volunteers exhibited strong systemic and mucosal immunity after three doses of potato were consumed [37]. These results demonstrated that plant-edible vaccines could be used in global immunization projects at a low cost. Subsequently, many other vaccine genes were expressed in a variety of crops, including lettuce, banana, and tomato fruits. Several plant vaccines are now in clinical trials that have produced encouraging data [38]. PMF production strategies and challenges, such as biosafety, appropriate expression systems, possible and potential applications, are discussed in the present review with the idea of demonstrating a feasible approach for the potential commercialization of a PMF product.

## 2. Advantages

A very advantageous aspect of PMF research is that it does not require a large financial investment to conduct initial studies. Plants can be grown in a greenhouse or even in a biosafety lab if required. The cost of plant maintenance for PMF is low, relative to *E. coli*, yeast, or mammalian cell expression systems (see Table 3); and the source (plant leaves or seeds) for making the recombinant protein is potentially unlimited [7,8,35]. Plant expression systems have several major advantages over prokaryotic and other eukaryotic cell systems in regards to production speed, cost, and safety. Plants can correctly fold and assemble complex proteins, such as secretory antibodies, full size immunoglobulins and the homodimeric vascular endothelial growth factor (VEGF). Human VEGF produced in barley grain has been commercialized for research use [39,40]. Plant-expressed human VEGF is used as treatment for thinning hair (UNICO Enterprises, Pasadena, CA, USA). Plants also have the capacity to introduce post-translational modifications. The use of plants also eliminates potential contamination of the therapeutic drug with animal pathogens (prions, viruses, and mycoplasmas), thus increasing safety. In general, the cost of PMF-derived products is only 0.1% of mammalian cell culture systems and 2%–10% of microbial systems.

**Table 3:** Comparison of different expression platforms for the production of pharmaceuticals (modified from Spök and Karner 2008 [41], European Communities).

Comparisons	Transgenic Plant	Plant Cell Culture	Bacteria	Yeast	Mammalian Cell Culture	Transgenic Animals
Overall cost	Very low	Medium	Low	Medium	High	High
Scale-up capacity	Very high	Medium	High	High	Very low	Low
Production scale	Worldwide	Limited	Limited	Limited	Limited	Limited
Protein yield	High	High	Medium	High	Medium-High	High
Protein folding accuracy	High	High	Low	Medium	High	High
Glycosylation	Minor differences	Minor differences	None	Incorrect	Correct	Correct
Product quality	High	High	Low	Medium	High	High
Contamination risks	Low	Low	Endotoxins	Low	Virus, Prions, oncogenic DNA	Virus, Prions, oncogenic DNA
Safety	High	Non-specific	Low	Unknown	Medium	High
Storage cost	Inexpensive	Moderate	Moderate	Moderate	Expensive	Expensive

## 3. Challenges

Current methods in plant biotechnology cannot precisely control the expression level of transgenes in plants in a consistent manner and not every plant species is readily transformed. This means that the amount of pharmaceutical produced may vary in each plant species, or even in different plant parts (*i.e.*, leaves, fruit, and seeds). Levels of expression in subsequent generations may also vary. Given this scenario, it is very difficult to accurately quantify the appropriate dosage of edible vaccines for children and adult patients. Edible vaccines can also trigger immune tolerance after oral administration. Lastly, most of the ingested protein will be degraded by digestive processes. Collectively, these disadvantages greatly restrict the clinical use of edible vaccines [30,32].

While the science of PMF is relatively new, microbial and animal cell expression systems have been used for over 30 years, and industry has developed standard and high-throughput purification protocols. In contrast, protocols for the purification of plant-derived pharmaceutical proteins are varied. While plants such as tobacco, maize, and rice, have been used for greenhouse or open-field production of PMF products, each plant species consists of unique sets of proteins and metabolites. As a result, each PMF platform requires its own purification protocol that is tailored to the product being generated and the plant production system [14,15,16,41,42,43]. Factors, such as plant phenolic compounds, plant pathogens, secondary metabolites, pesticides, and fertilizers, increase the difficulty of purifying a PMF product at an industrial level. Field crop-based PMF platforms, such as maize or rice, have pollen contamination issues which raise biosafety concerns as the pollen may contaminate non-transgenic crops that are part of normal agricultural production [44,45,46]. Currently, FDA has a restricted policy for using food crops for the production of recombinant pharmaceutical compounds [47].

ProdiGene, Inc. company (College Station, TX, USA) launched the large-scale production of transgenic maize that produces trypsin. However, the USDA discovered that plant remnants from a previous ProdiGene trial had contaminated a nearby conventional field. ProdiGene was fined $250,000 and charged $3 million to cover the cleanup operation of mishandling the field test. The punitive action forced ProdiGene into bankruptcy. This event represented a significant setback to the commercial use of PMF [48]. In order for PMF to succeed, both standard biosafety procedures and purification protocols need to be established. In this regard, tobacco is a very good candidate for PMF production since it is not a food crop and cannot contaminate other crops by the spread of transgenic pollen [41]. The procedure for gene transfer and expression in tobacco is also simple and well established. Transgenic tobacco plant can be produced in six months and it can produce the protein of interest in both leaves and seeds.

## 4. PMF Products for Use as Topical Applications and Health Supplements

Some reports have indicated that subcutaneous injections of plant-derived proteins could induce an immunogenic response to plant-specific glycans [49,50,51,52,53]. Topical applications of plant-derived glycoproteins in humans, however, have not resulted in any adverse effects and therefore represent a potential approach for PMF-based products. Topical application of a recombinant plant secretory antibody prevented oral *Streptococcal* colonization for at least four months in humans [54]. Topical application of soybean-derived monoclonal antibodies (mAbs) readily diffused in human cervical mucus and prevented herpes simplex virus 2 (HSV-2) infection [55]. Tan *et al.* (2014) [56] expressed human acidic fibroblast growth factor 1 (FGF-1) in the medicinal plant *Salvia miltiorrhiza*. The product combined the medicinal function of both FGF-1 and bioactive compounds within the medicinal plant. Topical application of extracts obtained from the transgenic medicinal plant significantly stimulated fibroblast cells, promoted blood vessel formation, and accelerated the healing process of burn wounds in mice. This is an example of how PMF can be used to combine the therapeutic function of a recombinant protein and the inherent properties of a medicinal plant. Topical application of a plant extract would significantly reduce the cost of purification and downstream processing. In general, topical application is safer than oral consumption or injection, which would help to address concerns about public safety.

A primary objective of PMF is to reduce the cost of producing novel therapeutic proteins. Using PMF to create a vegetable, seed or fruit health supplement could be a practical alternative to using PMF to develop a processed pharmaceutical drug. Guan *et al.* (2014) [57,58] expressed lumbrokinase, an anti-thrombotic enzyme from earthworm, in sunflower kernels. Mice and rats that were fed the transgenic kernels exhibited a strong degradation of blood clots [58]. Unlike a vaccine or a therapeutic protein, lumbrokinase has been widely sold and used as a health supplement to dissolve blood clots and maintain healthy cardiovascular function in people. This makes lumbrokinase a good candidate for PMF since, in general, health supplements do not need a medical prescription and have less regulations for commercialization [59].

## 5. PMF Production Platforms

### 5.1. Transient Expression Platform

**Figure 2:**
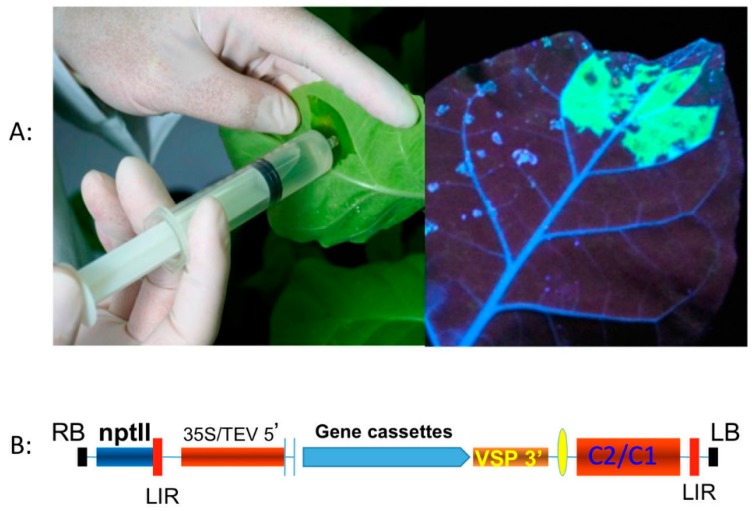
A plant-based transient expression system. Bean yellow dwarf virus (BeYDV) based single-vector DNA replicon system, pBY030.2R, is used to transiently express green fluorescent protein (GFP) in tobacco leaves. (**A**) Infiltration of *Agrobacterium* carrying the GFP transgene (**Left**); and transient expression of GFP (**Right**). Infiltrated leave examined with a UV lamp at four days post infiltration; (**B**) Diagram of the pBY030.2R vector (kindly provided by Hugh Mason, Arizona State University, Tempe, AZ, USA) used in this study. 35S/TEV 5′, CaMV 35S promoter with tobacco etch virus 5′ UTR; VSP 3′, soybean vspB gene 3′ element; npt II, expression cassette encoding *nptII* gene for kanamycin resistance; LIR, long intergenic region of BeYDV genome; SIR (yellow oval), short intergenic region of BeYDV genome; C1/C2, BeYDV ORFs C1 and C2, encoding *Rep* and *RepA*; LB and RB, the left and right borders of the T-DNA region.

It often takes six months to a year or more to produce transgenic plants. Several generations are required to generate plants that are homozygous for the transgene. Most transformation technologies also result in the gene being inserted randomly into the plant genome. The factor of random insertion, along with the need to identify regulatory elements (promoters) to drive a high-level of foreign gene expression, often results in a low yield of the recombinant protein even in plants that have been stably transformed. The time needed to utilize a standard PMF approach is unsuitable for addressing sudden viral epidemics, such as an outbreak of Ebola, severe acute respiratory syndrome (SARS), or MERS-CoV. Transient expression systems can be used as an alternative approach to produce recombinant protein within three to five days [60,61,62,63] (see Figure 2).

Various viral vectors have been developed for small- or medium-scale PMF production. For example, Mason *et al.* (2010) [64] developed a highly efficient, bean yellow dwarf virus (BeYDV) based single-vector DNA replicon system, which incorporated multiple DNA replicon cassettes. They were able to produce 0.5 mg of antibody per gram leaf (fresh weight) in tobacco leaves within four days following infiltration. Mapp Biopharmaceutical Inc., using similar methodology, transiently expressed the humanized antibodies, MB-003 and ZMab, in tobacco leaves. MB-003 and ZMab were later combined and designated as ZMapp. The use of these antibodies as a pharmaceutical drug was able to cure 100% of Ebola infected rhesus macaques primates [65]. *Agrobacterium* infiltration of leaves for transient expression is very lab intensive and is the primary reason that sufficient supplies of the Ebola vaccine are not available. In September 2014, the U.S Department of Health and Human Service (HHS) provided a grant of $42.2 million to Mapp Biopharmaceutical Inc. for developing a method for large-scale production of ZMapp. With further optimization of the transient expression system, large-scale production in a short time period (one week) may become feasible [66].

### 5.2. Bioreactor-Based Platforms

#### Plant-Cell-Culture System

Plant-cell-culture based bioreactors currently show more promise than traditional PMF using whole plants to produce pharmaceuticals [67,68,69]. Similar to microbial or mammalian cell bioreactors, plant cells are cultured in a sealed, sterilized container system without human pathogens or soil contamination. Biosafety concerns associated with the unintentional distribution of pollen and cross-fertilization are also eliminated. Cultured plant cells require only simple nutrients to grow, so the operational cost is much less expensive than mammalian or microbial bioreactors. Downstream purification and processing of the recombinant protein is less complicated in the absence of complex plant fibers and an array of secondary metabolites, which significantly reduces production costs. As previously noted, the first FDA approved PMF-based pharmaceutical, taliglucerase alfa, used to treat Guacher’s disease, was produced in carrot cell suspension cultures (Table 1 and Table 2) [17]. Since Gaucher’s disease is a very rare disease, treatment of this disease with an orphan drug is very costly ($200,000 US annually per patient for life). Using a carrot cell production system, however, reduces the cost to $150,000/patient/year. More than 20 recombinant proteins have been produced from plant cell culture systems [41]. The tobacco strains, BY-2 and NT-1, are the most popular plant cell-culture based strains used as bioreactors in PMF. The recombinant protein can be expressed to be secreted into the culture medium to simplify the downstream purification process. The pore size in plant cell walls, however, may prevent some foreign proteins from being secreted depending on their size and folded architecture [67]. Proteolytic activity in cultured cells may also result in a low yield of antibodies. Magy *et al.* [68] demonstrated that the proteolytic profile is host species specific. They tested the combination of the isotype, culture conditions, and host species and found that the best combination resulted in 10-fold differences in the expression level. More than 30 mg/L intact antibody was produced in optimum conditions. A yield of 20 mg/L of the human monoclonal antibody M12 was produced in tobacco-BY-2 cell cultures in a 200 L bioreactor [69]. A commercial, cost-effective plant cell culture platform, ProCellEx™ (Protalix Biotherapeutics, Karmiel, Israel) has been developed that significantly reduces costs for industrial scale recombinant protein production [70].

### 5.3. Moss Culture

Currently, the use of moss, a non-seed plant, is being investigated as a candidate for the production of pharmaceutical proteins in bioreactors [71,72,73]. The ability of moss cells to photosynthesize in culture significantly reduces the cost of culture nutrients. As in yeast and plant cell suspension cultures, recombinant proteins can be designed to be secreted into the culture medium in moss cultures, which facilitates downstream processing and purification of the recombinant protein. Using genetic manipulation, moss cells can also produce a humanized form of a glycosylated protein, lewis Y-specific mAb MB314 [72], which reduces concerns, as noted below, about plant-specific glycosylation. Some recombinant therapeutic proteins, such as Epidermal Growth Factor [74], α-galactosidase [74], α-amylase [75], a glyco-optimized version of the antibody IGN311 [76], a multi-epitope fusion protein from the human immunodeficiency virus [77], *etc.* have all been produced in moss cultures. Cultures of the moss, *Physcomitrella patens* (*P. patens*), are the most commonly used plant material within bioreactors to enable protein production. A German biopharmaceutical company, Greenovation Biotech GmbH, has developed a *P. patens*-based platform (Bryo-Technology) for large-scale, high quality, recombinant protein production. Examples include Moss-GAA for Pompe Disease, Moss-GBA for Gaucher’s Disease, and Moss-AGAL for Fabry Disease. Moss-AGAL has completed preclinical trials and is entering Phase I testing (Table 1).

### 5.4. Algal Bioreactors

Microalgae cultures have been used for biofuel and foreign protein production for many years [78,79,80]. Microalgae have a very simple structure, and can be unicellular, colonial, or filamentous. Algae can produce large amount of biomass within a very short period due to their short life cycle. The downstream purification of recombinant proteins in algae is similar to yeast and bacterial systems, and is therefore generally less expensive than whole plant production systems. However, recombinant proteins produced from algae do not undergo certain post translational modifications, and as a result, may not be suitable for the production of some glycoproteins. For example, algae may not be able to produce human forms of glycosylated proteins due to a lack of the proper enzymatic machinery [81]. However, a variety of therapeutic and diagnostic recombinant proteins, including vaccines, enzymes, and antibodies, have been produced in algal systems [82,83,84]. In some cases, however, foreign genes are only expressed transiently in algae [85,86,87]. Efficient and stable expression of foreign genes in algae is greatly improved by the use of strong promoters, proper codon usage, intron integration, and specific transformation methods [88]. An optimized microalgae production system has been developed by a USA-based algae bioreactor company, PhycoBiologics [24]. Recombinant protein yields up to 20% of total soluble protein have been obtained, which makes the algal platform a promising approach for the production of commercial pharmaceuticals [24].

### 5.5. Seed-Based Platforms

**Figure 3:**
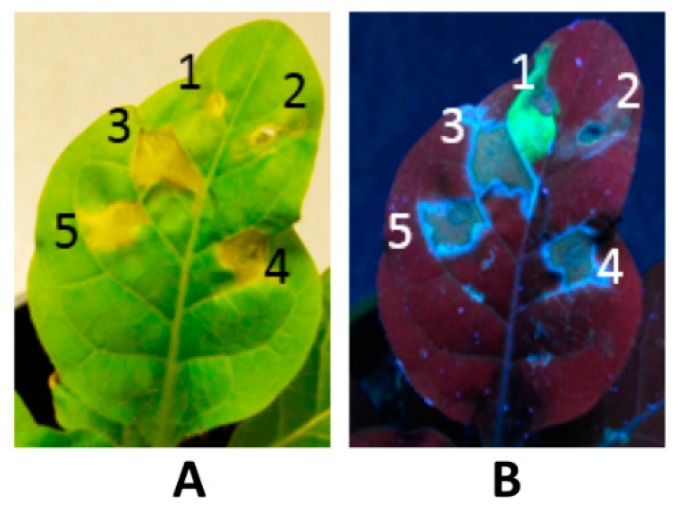
Production of some foreign proteins in leaf cells causes necrosis of plant leaf tissues: 1: GFP; 2: *Agrobacterium tumefaciens* only; 3: Nattokinase; 4: Lumbrokinase; and 5: DSPAα1 (vampire bat plasminogen activator α1). Picture shows leaf necrosis under visible (**A**); and UV light (**B**) four days after infilration using a transient expression platform.

Protein stability is also an important issue in the storage of harvested PMF-based recombinant products. Currently, most pharmaceutical proteins are synthesized in leafy crops for optimum biomass. Leaf proteins, however, are subject to rapid proteolytic degradation after they are harvested [41]. Long-term storage of leaf material is also very challenging. Overexpression of foreign proteins in leaf cells may also result in necrosis and subsequent cell cell death [89]. Our own prelimary studies demonstrated that transient expression of various blood clot-dissolving serine proteinases, such as vampire bat plasminogen activator (DSPAα1), nattokinase, and lumbrokinase, in leaves resulted in leaf necrosis four days after infiltration (Figure 3). When these proteins were targeted to in seeds, however, cell necrosis was not observed and the purified proteins exhibited the ability to dissolve fibrin and blood clots (unpublished data). Therefore, targeting the production of PMF-based products to seeds is becoming an attractive alternative [8,57,58,90,91,92,93,94].

Seed-based systems for PMF have been developed in various plant species, including *Arabidopsis* [90,92], tobacco [91], rice [93], and corn [94]. Recombinant proteins targeted to seeds have been reported to accumulate to very high levels. The use of a seed specific, *Phaseolus vulgaris* regulatory sequence to drive transcription of a murine single chain variable fragment (scFV) in *Arabidopsis* resulted in high yield of recombinant protein (36.5% of total soluble seed protein) [90]. The abundance of human lysozyme in transgenic rice grains was 1% of grain weight [95]. The level of scFV accumulation approached 25% of total protein in tobacco seeds when scFV was fused with elastin-like polypeptides [96].

Different subcellular compartments have been targeted for recombinant protein in order to increase protein stability in seeds. The ER (endoplasmic reticulum) compartment in plant cells has been demonstrated to contain few proteases and therefore represents a relatively protective environment. Retention of proteins in the ER therefore increases protein stability and yield due to the lack of protein degradation. KDEL, an ER signal peptide, was used to target the deposition of a recombinant protein to the ER [91,97]. Recombinant protein targeted to seeds also allows long-term storage (up to three years) at room temperature without a detectable loss in activity [8].

Targeting of PMF-based products to seeds allows for long-term protein stability, as well as easy harvesting, storage, and transportation. Relative to leaves, seeds contain fewer native proteins and less phenolic compounds and secondary metabolites. Seeds are easy to surface wash and sterilize which also facilitates commercial production. Collectively, the advantages of producing recombinant proteins in seeds make this platform a cost-effective platform for PMF-based products [8,98].

## 6. Humanized Glycosylation in Plants for “Glycan-Better” Products

Greater than 50% of human proteins are glycosylated. One third of all approved pharmaceuticals are glycoproteins [41]. Plants present an advantage for the production of recombinant proteins due to their capability of performing a variety of post-translational modifications, including glycosylation and lipid addition. Plants and animals share a similar early stage glycosylation pathway before nascent *N*-glycan reaches the Golgi apparatus. This pathway produces β-(1,4)-linked galactose and sialic acid in animals and *N*-glycan (core β-(1,2) xylose and core α-(1,3)) fucose moieties in plant. Glycosylation of native plant proteins is essential for their proper function during plant growth and development [99,100,101].

Plant-specific, hyperglycosylated proteins, however, may be immunogenic in humans. Immunization of mice and rats with horseradish peroxidase elicited the production of antibodies (Abs) specific for plant glycans [52]. Immunization of rabbits with a plant-derived, human monoclonal antibody resulted in a strong immune response to a plant specific glycan [49]. A human sera test also detected antibodies to plant glycans. The anti-plant glycan immune response is highly undesirable and could prevent regulatory approval of a glycosylated PMF when the recombinant protein is intended to be administered by injection [11]. Few human trials have investigated whether or not PMF-based recombinant proteins can elicit an immunogenic response in human patients, although it has been estimated that at least 20% of patients would be allergic to plant specific *N*-glycan [50]. Even though no clinical trials have revealed an adverse effect from an immune response to plant glycans, the subject is still a source of debate and has hampered the development of PMF-based pharmaceuticals [11,102].

Researchers have addressed concerns about plant-specific glycosylation by altering the pathway that plant cells use to process the recombinant protein. Recombinant proteins have been targeted to the endoplasmic reticulum (ER) where non-immunogenic, high-Man-type *N*-Glycans are produced [103]. Other studies have tried to prevent the production of plant glycan moieties using a knockout [104] or by silencing α-(1,3) FucT and β-(1,2) XylT with RNAi or an antisense approach [79,105,106]. Strasser *et al.* (2004) [104] produced a triple knockout in *Arabidopsis*, resulting in the absence of plant-specific glycans, in order to produce recombinant protein with humanized glycan structures. A recombinant human antibody fragment and an active enzyme were successfully produced with a controlled glycosylation pattern using this triple knockout *Arabidopsis* mutant [99]. RNAi lines of *Lemna* [79], alfalfa [105], and tobacco [106] have been developed that down regulate β-(1,2) XylT and α-(1,3) FucT activity. All of these RNAi lines were capable of producing human proteins without the addition of plant-specific glycans.

Efforts to optimize the plant glycosylation pathway have improved the therapeutic safety of PMF-derived recombinant proteins and reduced concerns about plant-specific glycan immunogenicity [103]. Protalix Biotherapeutics has produced a “glycan-better” taliglucerase alfa (ELELYSO^TM^) to treat the rare genetic disorder, Gaucher’s disease. The enzyme is targeted to the vacuole of carrot cells. Unlike the equivalent product produced in CHO cells, which express a recombinant protein with a terminal sialic residue that needs to be removed by an exoglycosidase to expose the terminal mannose moiety, the recombinant enzyme produced in a plant-based system already exhibited terminal mannose residues capable of specifically binding to the receptor on a microphage. The plant derived recombinant protein does not need an enzyme to expose mannose residues, which significantly lowers the cost and complexity of downstream processing; thus reducing the costs of production and therapy. Another example is *Lemna* (duckweed)-derived mAbs (Biolex/Synthon, Durham, NC, USA). RNAi technology was used to reduce the level of enzymes involved with plant core β-(1,2) xylose and core α-(1,3) fucose synthesis The recombinant mAbs produced in the duckweed system contains a single major *N*-glycan that has a 20-fold better antibody-dependent cell-mediated cytotoxicity and a 10-fold higher cell receptor binding activity than mAbs produced in CHO cells [79].

## 7. Downstream Processing

Extraction and purification of PMF-derived pharmaceutical proteins can be complex and costly from an industrial perspective. It has been estimated that purification and downstream processing represents 80% to 90% of the cost of producing PMF-derived pharmaceuticals [42]. For each platform, a specific recovery and purification protocol has to be optimized. Numerous protein recovery and purification processes have been developed on a case-by-case basis [107]. For example, Kentucky Bioprocessing Inc. (Owensboro, KY, USA) has developed a platform of protein expression, production, and processing in transgenic tobacco that conforms to Current Good Manufacturing Practices (cGMP) [23]. Healthgen Biotechnology Corp. (Wuhan, China) has developed an rice seed- based platform, OryzExpress, for producing a variety of products, such as recombinant human albumin, antitrypsin, protease inhibitor, IGF-1, *etc.* [29]. Plant substances, such as waxes, phenolic compounds, pigments, lignin, and endogenous proteases, also create problems in downstream processing. For instance, phenolic oxidation can result in protein aggregation and precipitation, or endogenous proteases can cause proteolysis [88]. The overall strategy used for downstream processing has to be economically competitive, robust, and compliant with cGMP [108]. In 2015, Caliber Biotherapeutics [109] built a plant-based manufacturing facility that has capacity to process over 3500 kg of tobacco (*Nicotiana benthamianai*) biomass per week. A transient expression approach is used to produce “biobetter” monoclonal antibodies (mAb) and anti-viral mAb. The downstream processes in this facility include an automated system for the production of various buffers used for separation, high-capacity chromatography, and formulation (cryostorage). Yields of the recombinant protein are in the range of 62%–68% of total protein and the purity of the final product is 95%–98% [110]. The costs of producing, purifying, and formulating the PMF products has been significantly reduced. For example, plant-derived vaccines for the flu cost $0.10 to $0.12 per 50-μg dose [110].

## 8. Conclusions

Plants have the potential to rapidly produce recombinant proteins on a large scale at a relatively low cost compared to other production systems. PMF-based production of pharmaceuticals, topical compounds, and nutritional supplements is feasible, however, concerns about biosafety, human health (allergenic response to plant-specific glycans), and other factors need to be adequately addressed. Downstream processing and purification of PMF products is currently tedious and costly. Systems need to be developed that simplify the purification process in order to make the production of industrial quantities of PMF-based products feasible and cost effective. The right candidate genes, a strong commercial need, and a good production system will build a bridge between basic research on PMF and its commercial application.

## Abbreviations

Plant Molecular Farming (PMF), Single chain variable fragment (scFV), Endoplasmic reticulum (ER), Bean yellow dwarf virus (BeYDV).
